# Can Oscillatory Alpha-Gamma Phase-Amplitude Coupling be Used to Understand and Enhance TMS Effects?

**DOI:** 10.3389/fnhum.2019.00263

**Published:** 2019-07-31

**Authors:** Johanna Wagner, Scott Makeig, David Hoopes, Mateusz Gola

**Affiliations:** ^1^Swartz Center for Computational Neurosciences, Institute for Neural Computation, University of California, San Diego, San Diego, CA, United States; ^2^Department of Radiation Medicine and Applied Sciences, School of Medicine, University of California, San Diego, San Diego, CA, United States; ^3^Institute of Psychology, Polish Academy of Sciences, Warsaw, Poland

**Keywords:** transcranial magnetic stimulation, TMS, phase-amplitude coupling, PAC, neurostimulation, oscillations, EEG

## Abstract

Recent applications of simultaneous scalp electroencephalography (EEG) and transcranial magnetic stimulation (TMS) suggest that adapting stimulation to underlying brain states may enhance neuroplastic effects of TMS. It is often assumed that longer-lasting effects of TMS on brain function may be mediated by phasic interactions between TMS pulses and endogenous cortical oscillatory dynamics. The mechanisms by which TMS exerts its neuromodulatory effects, however, remain unknown. Here, we discuss evidence concerning the functional effects on synaptic plasticity of oscillatory cross-frequency coupling in cortical networks as a potential framework for understanding the neuromodulatory effects of TMS. We first discuss evidence for interactions between endogenous oscillatory brain dynamics and externally induced electromagnetic field activity. Alpha band (8–12 Hz) activities are of special interest here because of the wide application and therapeutic effectiveness of rhythmic TMS (rTMS) using a stimulus repetition frequency at or near 10 Hz. We discuss the large body of literature on alpha oscillations suggesting that alpha oscillatory cycles produce periodic inhibition or excitation of neuronal processing through phase-amplitude coupling (PAC) of low-frequency oscillations with high-frequency broadband (or gamma) bursting. Such alpha-gamma coupling may reflect excitability of neuronal ensembles underlying neuroplasticity effects of TMS. We propose that TMS delivery with simultaneous EEG recording and near real-time estimation of source-resolved alpha-gamma PAC might be used to select the precise timing of TMS pulse deliveries so as to enhance the neuroplastic effects of TMS therapies.

## Introduction

Non-invasive transcranial magnetic stimulation (TMS) of the human brain has gained increasing popularity over the last decades and today is being widely used in both research and clinical applications. In TMS, brief, high-intensity electromagnetic pulses are produced in one or more wire coils (transducers) placed tangential to the scalp, inducing electrical currents in the underlying brain area. TMS can be applied as single, isolated pulses or as trains of stimuli [termed repetitive or rhythmic TMS (rTMS)], producing effects on the brain that can accumulate with repeated exposure and outlast the course of treatment (Rossi and Rossini, 2004; Ridding and Rothwell, 2007; Thut and Pascual-Leone, 2010). The effects of rTMS on brain activity can be observed near the site of maximal cortical stimulation as well as at anatomically remote but functionally connected cortical and subcortical areas (Strafella et al., 2001; Pogarell et al., 2006; Tik et al., 2017) suggesting that rTMS may modulate the dynamics of affected brain circuits (Medaglia et al., 2017; Vöröslakos et al., 2018). Possible long-lasting neuromodulatory effects of rTMS on brain circuits are of great interest in the clinical therapeutic arena, as they are thought to have potential benefit for a wide range of neurological and psychiatric pathologies thought to be characterized by disturbance in functional connectivity among brain regions (for reviews, see Schnitzler and Gross, 2005; Uhlhaas and Singer, 2010).

The precise mechanisms by which TMS exerts its neuromodulatory effects remain unknown. Research in humans suggests that both immediate and longer-term effects of rTMS are mediated by the interaction of the induced electrical current with endogenous oscillatory dynamics (Klimesch et al., 2003; Thut et al., 2011). These studies show that targeting subject- and task-specific oscillatory frequency bands can increase the effects on oscillatory band power as measured by scalp electroencephalography (EEG) and also, subsequent cognitive and behavioral performance (Klimesch et al., 2003; Thut et al., 2011; Veniero et al., 2011; for reviews, see Bergmann et al., 2016; Thut et al., 2017; Hanslmayr et al., 2019). Timing TMS pulse presentations to specific phases of ongoing oscillatory activity has also been demonstrated to increase subsequent corticospinal excitability—a measure of cortical plasticity (Bergmann et al., 2012; Zrenner et al., 2018). This suggests that electric field activity in the brain produced by TMS pulses may enhance underlying cortical excitability which is modulated by oscillatory field activity occurring within distributed brain networks, thereby affecting basic synaptic mechanisms producing long-term potentiation (LTP) and/or depression (LTD) within those networks (Ridding and Rothwell, 2007).

Here, we first review evidence for interactions between externally induced brain electric field potentials and endogenous cortical field activity. We then discuss how high and low excitability states relate to the phase of oscillatory field potentials, and how phase controls excitability states through cross-frequency coupling. Based on this body of research we argue that brain stimulation timed to particular phases of spontaneous low-frequency oscillations may enhance neural excitability, by increasing occurrence of appropriately timed high-frequency gamma oscillations through the mechanism of cross-frequency phase-amplitude coupling (PAC). The neurophysiological properties of oscillatory coupling may explain oscillatory-phase-guided rTMS neuroplasticity effects and thereby help to identify high-excitability phases to best target with TMS.

## Basic Understanding of Interactions Between Endogenous Oscillations and Externally Induced Brain Electric Field Activity

Direct evidence for interaction between externally-induced brain electric field activity and endogenous cortical oscillations comes from recordings of local field potentials and multiunit activity in animals. *In vitro* recordings in animal brain slices have shown that applied weak oscillatory electric fields affect the transmembrane voltage of nearby neurons, biasing neuronal spike timing (Anastassiou et al., 2011; Anastassiou and Koch, 2014). Fröhlich and McCormick (2010) induced weak sinusoidal currents *in vitro* leading to concentrated bursts of neural firing in affected neuropile in the applied current low-frequency oscillation pattern. This occurred for levels of induced current well below those needed to increase the net firing rate of the involved neurons, but comparable to levels of *in vivo* endogenous local field potential in the same tissues.

Results of Ali et al. (2013) suggest that matching stimulation frequency to endogenous brain activity is a crucial requirement for weak oscillatory electric fields to have an effect on network dynamics since the depolarization caused by a weak supplied electric field is too small to activate neurons at rest. Weak electric fields applied at the endogenous oscillation frequency may enhance endogenous oscillations but fail to induce a frequency shift when the stimulation frequency is not matched to the endogenous oscillation (Schmidt et al., 2014). In essence, neurons need to be close to their firing threshold for a stimulation-induced sub-millivolt perturbation in membrane voltage to effectively modulate endogenous network neural spiking statistics and affect brain network dynamics.

Research in humans has shown that rTMS tuned to endogenous EEG oscillations enhances cortical oscillations in the targeted band and may also produce behavioral changes (Sauseng et al., 2009b; Romei et al., 2010). Thut et al. (2011), for example, reported phase-locking of EEG activity to magnetic pulse trains of participant- and task-specific alpha-frequency rTMS. Entrained rTMS-evoked EEG activity may also outlast the stimulation, suggesting that an endogenous, rTMS-induced mode of brain activity has been produced by the stimulation (Hanslmayr et al., 2014). As reported by Klimesch et al. (2003), increased alpha power following rTMS pulse trains delivered at a subject’s individual alpha frequency was associated with a significant improvement in subsequent performance of a mental rotation task.

Other research supports the concept that ongoing oscillations create periodic “windows of excitability” that can be targeted with TMS. Dugué et al. (2011), for example, showed that the phase of ongoing (8–12 Hz) alpha oscillations, within the 400 ms before a TMS pulse applied over visual cortex, significantly co-varied with the pulse-induced visual illusions (phosphenes). Similar observations have been described in the sensorimotor system for which the most dominant oscillatory frequency is the (8–12 Hz) mu rhythm. Zrenner et al. (2018) triggered TMS pulse triplets (three pulses at 100 Hz) at varying intervals (longer than 0.75 s) targeted to occur at negative or positive peaks of healthy participants’ spontaneous EEG mu rhythms. Only stimulation at the surface negative peak of the mu-rhythm cycles resulted in a LTP like increase in corticospinal excitability [as measured by subsequent increase of the motor-evoked potential (MEP) amplitude]. Bergmann et al. (2012) triggered single-pulse TMS over the primary motor cortical hand area within EEG relative (surface-negative) “up-states” and (surface-positive) “down-states” during sleep. Both TMS-evoked and subsequent MEPs were consistently larger when stimulation occurred during slow oscillatory (negative-going) up-states than during (surface-positive) down-states. These results can be explained by direct effects of local field activity on neural excitability, including little understood ephaptic (non-synaptic) effects on the intra-neuronal environment (Fröhlich and McCormick, 2010; Anastassiou et al., 2011; Anastassiou and Koch, 2014). This work raises the intriguing possibility that real-time information on current brain state derived from EEG recording can be used to maximize TMS induction of cortical plasticity in humans.

## Evidence for Cortical Excitability States Changes with Alpha Oscillatory Cycles

Understanding how cortical excitability is affected by endogenous local field potentials, therefore, seems crucial to further development and optimization of TMS stimulation protocols. As outlined above, oscillations in local cortical field potentials are now seen to both reflect and induce cyclical variation in the excitability of involved cortical neuronal ensembles (Bishop, 1933; Freeman and Rogers, 2002; Vanhatalo et al., 2004), making them more likely to fire in one phase of the cycle than in another (Klausberger et al., 2003; Haider and McCormick, 2009; Canolty and Knight, 2010; Canolty et al., 2010). Targeting oscillations in the (8–12 Hz) alpha frequency band is of special interest, as most current clinical TMS protocols involve some form of stimulation in this frequency range. For example, 10-Hz rTMS over frontal brain areas has proven to have therapeutic benefit in treatment-resistant depression; accordingly, most rTMS protocols approved to date by the United States Food and Drug Administration (FDA) involve 10-Hz stimulation (O’Reardon et al., 2007; George et al., 2010; Perera et al., 2016).

There is growing evidence that 8–12 Hz posterior alpha and sensorimotor (mu) oscillations play a significant role in modulating brain information processing in humans by providing a periodic inhibitory influence within their generator regions (Klimesch et al., 2007; Jensen and Mazaheri, 2010; Mathewson et al., 2011). Recent findings suggest that mu rhythms exercise strong inhibitory influence on local neuronal spike timing firing rate. Haegens et al. (2011) reported a rhythmic relation between mu-rhythm oscillations in monkey sensorimotor cortex and neuronal spiking, with neuronal firing highest at the (surface-negative) trough of the mu-rhythm cycle. Ai and Ro (2014) demonstrated that humans’ ability to perceive a weak tactile stimulus was predicted by the mu phase angle at stimulus onset in the EEG, suggesting that sensorimotor mu rhythms wield a strong inhibitory control on tactile perception.

A similar relationship seems to hold for alpha oscillations in the visual cortex. Mercier et al. (2015) for example, showed using ECoG data that reaction times are faster when local auditory and visual cortical theta/low alpha rhythms (5–8 Hz) are both in phase with the onset of an audiovisual stimulus. Other studies demonstrated that both phase and power of pre-stimulus alpha oscillations affect visual detection (van Dijk et al., 2008; Busch et al., 2009; Mathewson et al., 2009). Visual discrimination ability decreases with an increase in pre-stimulus alpha power (van Dijk et al., 2008) while detection performance for attended stimuli fluctuates in time with the pre-stimulus phase of spontaneous alpha oscillations (Busch and VanRullen, 2010). This phasic modulation of detection performance increases with stronger alpha entrainment to a rhythmic stimulus presentation (Spaak et al., 2014). Other research demonstrates that the phase of EEG alpha rhythm over posterior brain regions can reliably predict both stimulus-elicited cortical activation levels and subsequent visual detection (Mathewson et al., 2009). As well, blood oxygenation-level-dependent (BOLD) responses to brief fixation events have also been shown to vary as a function of the alpha phase of EEG independent component effective source processes (Scheeringa et al., 2011).

Research also shows that alpha oscillations influence the temporal resolution of perception. Two briefly presented visual stimuli may be perceived as a single stimulus or as two separate stimuli depending on whether they fall in one or two separate alpha cycles depending on the frequency of the alpha oscillation (Samaha and Postle, 2015). These and related findings (Varela et al., 1981; Zauner et al., 2012) have led to the conclusion that the frequency of the alpha cycle indexes the duration of “perceptual windows” (e.g., during the surface-negative phase of the alpha cycle), and controls variation in both the sensitivity and temporal resolution of visual perception (for reviews, see Hanslmayr et al., 2011; Mathewson et al., 2011; Shapiro et al., 2017).

Note that the direction of the relationship between cortical surface negative and positive peaks is ambiguous for sulcal rhythms since the polarity of alpha negative/positive peaks depends on the orientation of the vortical source patch in relation to the cortical surface. In addition, many of the above cited studies have analyzed EEG channel data (Mathewson et al., 2009; Busch and VanRullen, 2010; Samaha and Postle, 2015; Zrenner et al., 2018) which adds additional ambiguity in terms of oscillatory phase. By the broad spread of brain volume conduction, each EEG channel signal sums potentials from many effective brain sources (for example, see Makeig et al., 2004; Onton et al., 2005, 2006; Brunner et al., 2016). This needs to be considered when targeting alpha negative/positive peaks with TMS. Source-resolved estimation of EEG oscillatory phase, for example using Independent Component Analysis (Bell and Sejnowski, 1995; Makeig et al., 1996), can help to resolve this ambiguity.

Nevertheless, the here discussed studies suggest that mu and alpha rhythm cycles constrain neural spikes into occurring during brief time windows, leading to periodic suppression of neural processing with cortical surface negative and positive peaks in the mu/alpha cycle representing high and low excitability states respectively. Zrenner et al. (2018) provide deterministic evidence for this long-held belief, proposed by Elbert and Rockstroh (1987).

## Relationship to Cross-Frequency Phase-Amplitude Coupling of Neuronal Oscillations

What are the underlying functional mechanisms by which oscillatory phase changes the excitability of the local cortical area and state? Nested hierarchical cross-frequency PAC of cortical potentials, wherein phase in lower frequency bands modulates amplitude in respectively higher bands, has been proposed as a general mechanism supporting the encoding, storage, and retrieval of information in neural networks (Schroeder and Lakatos, 2009; Canolty and Knight, 2010; Fell and Axmacher, 2011; Bergmann and Born, 2018; Reinhart and Nguyen, 2019). Slow oscillations consist of alternating states of synchronized depolarization (up-state) and hyperpolarization (down-state) that propagate throughout the cortex, also reaching the thalamus *via* cortico-thalamic projections. Note that cortico-thalamic feedback may play a key role in the temporal control of cortical excitability by mediating phase alignment of neuronal firing and slow oscillatory peak depolarization.

The most-studied example of PAC is theta-gamma PAC in the hippocampus and cortex during working memory, information encoding, and retrieval (Fell and Axmacher, 2011) that is linked to theta phase-dependent processes of synaptic potentiation and depotentiation (Huerta and Lisman, 1995). It is hereby assumed that the phase of these spontaneous low-frequency oscillations control the excitability of local cortical neuronal ensembles, making them more likely to fire (Klausberger et al., 2003; Haider and McCormick, 2009; Canolty and Knight, 2010). This results in a systematic enhancement of responses to events occurring during high-excitability phases concurrent with broadband (30–200 Hz) gamma oscillatory bursts in cortical recordings, and suppression of responses to events occurring during low-excitability phases (Large and Kolen, 1994; Lakatos et al., 2005, 2008). Broadband gamma (30–200 Hz) activity has been suggested to reflect and index local neuronal population activity (Miller et al., 2009, 2014) indicating a state of high neuronal excitability (Fries et al., 2007).

**Figure 1:**
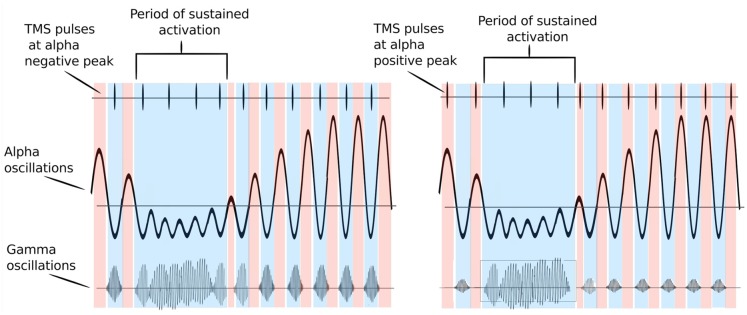
Possible mechanism of how alpha oscillations act on gating neural excitability: red color periods indicate periods of inhibition (alpha positive peak), while blue color indicates periods of activation (alpha trough). Transcranial magnetic stimulation (TMS) pulses are represented by vertical lines in the upper part of the figure. The bursts of gamma at each alpha cycle trough represent windows of neuronal processing. Left Panel: TMS pulses are delivered at alpha troughs. They thus coincide with gamma bursts that are coupled to alpha troughs, enhancing them. If TMS pulses arrive during high excitability phases at alpha troughs (blue colored periods), they occur simultaneously with gamma bursts and are able to enhance local brain processing. Right Panel: TMS pulses are delivered at alpha positive peaks. They thus occur at periods of relative inhibition when gamma bursts are absent and no enhancement of neural activity may occur. When alpha oscillations are sufficiently suppressed neurons can fire freely and TMS pulses delivered during this period can enhance gamma bursts irrespective of the phase of alpha oscillations. Adapted from Osipova et al. (2008) and Jensen and Mazaheri (2010).

Studies have demonstrated that timing of gamma bursts in the EEG is commonly modulated by alpha phase (Osipova et al., 2008; Voytek et al., 2010; for a review, see Canolty and Knight, 2010). The alpha cycle supposedly acts here as periodic inhibition—gamma bursts occur only during the cortical surface-negative troughs of the alpha cycle, and when the amplitude of alpha oscillations is sufficiently low. The strength of this relationship may change with movement or other cortical activation states (see Figure 1). A recent study (Herring et al., 2019) has provided deterministic evidence for the modulation of stimulus-induced gamma-band oscillations through alpha oscillatory phase. The authors applied weak alternating currents at subject’s individual alpha frequency ±4 Hz to the occipital cortex to mimic the functional effects of periodic inhibition during spontaneous alpha oscillations. The authors found that in fact the induced currents rhythmically suppressed visual stimulus-induced gamma-band power. The degree of gamma-band suppression predicted the reduction in visual detection performance, suggesting a direct modulation of cortical excitability by rhythmically shifting the neurons’ membrane potential. The here outlined ideas are supported by research showing that 10-Hz rTMS strengthened alpha–gamma cross-frequency phase synchrony and predicted changes in task accuracy in a visual working memory task (Hamidi et al., 2009).

Many EEG and ECoG studies show that a decrease in mu power in motor cortices is related to increased activation of the cortical area (Pfurtscheller et al., 1997; Crone et al., 1998; Pfurtscheller and Lopes da Silva, 1999; Miller et al., 2007). During movement as well as other activation states known to transiently block mu rhythm amplitude (Pfurtscheller and Neuper, 1997; Crone et al., 1998), alpha-gamma PAC may be diminished or eliminated (as also shown for beta-gamma coupling by Miller et al., 2007), and gamma bursts may occur freely throughout the alpha cycle. Other studies investigating the relationship between corticospinal excitability (as measured with MEPs) and alpha power showed that MEPs are larger when pre-stimulus mu power is lower (Zarkowski et al., 2006; Sauseng et al., 2009a), and pre-stimulus gamma power is higher (Zarkowski et al., 2006). Sauseng et al. (2009a) also showed that this effect was specific for local EEG alpha activity at sites overlying the cortical motor areas to which the TMS pulses were applied (as verified using source localization).

Thus, during a cortical activation state where alpha/mu power is suppressed, TMS pulses delivered at any phase of mu/alpha cycles may increase neuronal firing thus increasing subsequently cortical excitability. Instead during periods of increased mu/alpha power TMS pulses may best be delivered during surface negative alpha troughs to increase cortical activation states to be most effective.

## Conclusion

The studies discussed above suggest that mechanisms of PAC in local cortical brain field activities, the most prominent of which may dominate scalp EEG signals, could be exploited as a tool for more efficient TMS stimulation by incorporating information on the timing of neuronal excitability states.

Clinical TMS therapy has not changed much over the last 30 years with similar treatment protocols applied across different patient groups and a variety of disorders. One of the main practical issues in TMS therapy is that TMS after-effects are notoriously inconsistent, the same stimulation protocol inducing neural plasticity effects in opposite directions (Müller-Dahlhaus et al., 2008; Ziemann and Siebner, 2015). Bergmann et al.’s (2012) and Zrenner et al.’s (2018) studies provide insight into how stimulation protocols can be improved by increasing neuroplasticity through timing TMS pulses to oscillatory high excitability phases. These results were obtained in the motor cortex, however high and low excitability oscillatory phases likely differ over brain areas and frequency bands. This raises the question of how we can reliably identify high excitability phases of oscillations to target with TMS. Estimation of PAC may help to determine which exact phase of a given oscillation in the target brain area has the highest excitability. Methods for the estimation of event-related and time-resolved PAC (Voytek et al., 2013; Martínez-Cancino et al., 2019) may be either implemented before TMS stimulation or integrated into a real-time system to adapt timing of TMS pulses. Real-time estimation of PAC during TMS stimulation might be used to index of neuroplasticity and help determine the efficiency of the stimulation or predict the success of TMS therapy.

## Author Contributions

JW, MG and SM developed the concept for the manuscript. JW did the literature research and drafted the manuscript. MG, SM and DH provided comments to improve the manuscript.

## Conflict of Interest Statement

The authors declare that the research was conducted in the absence of any commercial or financial relationships that could be construed as a potential conflict of interest.
